# Lobectomy versus stereotactic ablative radiotherapy for medically operable patients with stage IA non‐small cell lung cancer: A virtual randomized phase III trial stratified by age

**DOI:** 10.1111/1759-7714.13103

**Published:** 2019-05-23

**Authors:** Young‐Seok Seo, Hak Jae Kim, Hong Gyun Wu, Sun Mi Choi, Samina Park

**Affiliations:** ^1^ Department of Radiation Oncology ChungBuk National University Hospital Chungcheongbuk‐do Republic of Korea; ^2^ Department of Radiation Oncology Seoul National University Hospital Seoul Republic of Korea; ^3^ Department of Radiation Oncology Cancer Research Institute, Seoul National University College of Medicine Seoul Republic of Korea; ^4^ Division of Pulmonary and Critical Care Medicine, Department of Internal Medicine Seoul National University Hospital Seoul Republic of Korea; ^5^ Department of Thoracic and Cardiovascular Surgery Seoul National University Hospital Seoul Republic of Korea

**Keywords:** Lobectomy, Markov model, non‐small cell lung cancer, randomized trial, stereotactic radiotherapy

## Abstract

**Background:**

Although the choice between stereotactic ablative radiotherapy **(**SABR) and lobectomy for early‐stage non‐small cell lung cancer (NSCLC) has been debated for years, the two procedures have not yet been directly compared in a randomized trial. We conducted a virtual randomized phase III trial stratified by age to compare the effectiveness of lobectomy and SABR for medically operable patients with stage IA (AJCC eighth) NSCLC using the Markov model analysis.

**Methods:**

A Markov model was developed to simulate a cohort of patients aged 45–85 years with stage IA NSCLC who had undergone either lobectomy or SABR and were followed up for their remaining lifetime. Each virtual patient was randomly assigned to undergo lobectomy or SABR, and 10 000 patients were allocated to each group. All estimates of the variables were obtained by a systematic review of published articles.

**Results:**

The lobectomy group showed a better life expectancy than the SABR group, in patients under 75 years of age. However, no statistically significant difference was seen in patients 75 years or older. The predicted life expectancy was 9.43 and 8.70 years in 75‐year‐old patients in the lobectomy and SABR groups, respectively. However, the 95%CI for the difference in life expectancy between the two groups was ‐ 0.06–1.50 years (*P* = 0.0689).

**Conclusions:**

The Markov model showed no statistically significant difference in the expected overall survival in stage IA NSCLC patients who were older than 75 years and had undergone SABR or lobectomy.

## Introduction

Lobectomy with sampling or dissection of mediastinal lymph nodes is the standard of care for early‐stage non‐small cell lung cancer (NSCLC). In the past decade, stereotactic ablative radiotherapy (SABR) has emerged as the preferred treatment for medically inoperable and operable early‐stage NSCLC.^1^, ^2^, ^3^, ^4^, ^5^, ^6^, ^7^, ^8^ Compared with conventional radiation therapy, SABR delivers ablative doses of radiation (biologically effective dose [BED] >100 Gy), in fewer fractions as highly conformal beams with the goal of delivering radiation to the tumor while sparing nearby, crucial, normal structures from radiation‐induced damage. Findings from retrospective, phase II prospective, population‐based studies and propensity‐matched analyses suggest that the overall survival after SABR is similar to that after surgery in patients with operable stage I NSCLC.^3^ With three previous trials (the STARS trial [NCT00840749], the ROSEL trial [NCT00687986], and the ACOSOG Z4099 trial [NCT01336894]) closing early because of slow accrual, SABR has not yet been directly compared with standard surgery in a randomized trial. Despite the small patient sample size and a short follow‐up, the pooled analysis of the STARS and ROSEL trials reported the estimated overall survival at three years to be 95% for the SABR group compared with 79% for the surgery group (hazard ratio [HR] 0.14 [95% CI 0.017–1.190], log‐rank *P* = 0.037).^3^ In the situation that investigators still question whether SABR can replace surgery for operable patients with early‐stage NSCLC, additional randomized studies comparing the two procedures are warranted. The existing literature on retrospective or randomized trials comparing surgery and SABR have overlooked age as an important prognostic factor. While mortality rates associated with surgery increase with age, those related to SABR are more affected by tumor size and the dose of radiation.^1^, ^2^, ^5^, ^8^, ^9^, ^10^, ^11^, ^12^, ^13^ Lobectomy would be a preferred strategy in young patients, whereas SABR would be preferable in older patients and in those with small tumors. To determine the optimal cutoff age after which SABR can be beneficial, a randomized study stratified by age is needed, which is practically impossible to perform in the clinical field. To overcome this practical limitation, we conducted a virtual randomized phase III trial in patients stratified by age, to compare the effectiveness of lobectomy and SABR for medically operable patients with stage IA (AJCC eighth) NSCLC using the Markov model analysis. A Markov model is a computerized model used to simulate the effects of competing interventions and identify key variables that affect the outcome of therapeutic strategies and the conditions that make one approach preferable to the other. A Markov model is widely used in the cost‐effectiveness analysis. Markov model analysis has the advantage that the prognostic factors such as tumor factors, performance status, the comorbidity, etc. are the same in both groups and only the effect of treatment modality can be compared.

## Methods

### Computerized simulation

**Figure 1:**
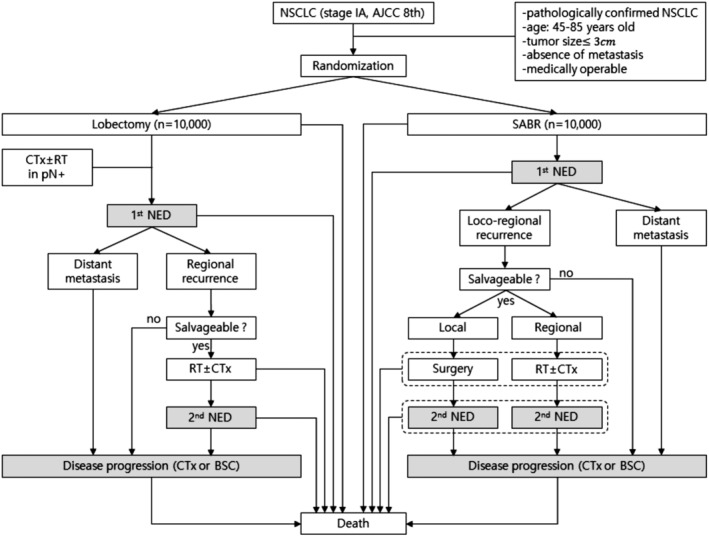
A Scenario for the Markov state transition model of NSCLC less than 3 cm.Each rectangle represents a state of health. From the initial state, patients are randomized to undergo lobectomy or SABR. Straight arrows represent the changes that may occur during each cycle or a very short time interval. In contrast, gray rectangles mean that the patients may remain in the same Markov state for more than one cycle.NSCLC, non‐small cell lung cancer; SABR, stereotactic ablative radiotherapy; CTx, chemotherapy; RT, conventional radiotherapy; pN+, pathologically positive lymph node; NED, no evidence of disease; BSC, best supportive care.

A Markov cohort model was developed to simulate a cohort of patients aged 45–85 years with stage IA NSCLC who had undergone either lobectomy or SABR and were followed‐up over a time period of their remaining life expectancy (Fig 1). Inclusion criteria were as follows: (i) pathologically confirmed NSCLC, (ii) tumor size≤3cm, (iii) absence of metastasis, and (iv) medically operable. Each virtual patient was randomly assigned to undergo lobectomy or SABR and 10 000 patients were allocated to each group.

For this Markov model, 22 states of health were defined, nine for patients undergoing lobectomy and 13 for those treated with SABR. From the initial state, patients were randomized to undergo lobectomy or SABR (Fig 1). For each state of health, the probability of transition to another state was determined based on the values extracted from the literature (Table 1). In two Markov states namely the disease‐free state and a state of disease progression, the patients could stay longer than one cycle. The cycle length of the model was set to be one year. A half‐cycle correction was used under the assumption that each transition happened halfway during the cycle. The Markov cycle was assumed to be repeated for 15 cycles. A commercially available software product (TreeAge Pro; TreeAge Software, Williamstown, MA) was used to generate the Markov model.

**Table 1:** Estimated values of the variables used for the Markov model extracted from the literature

Variables		Lobectomy	SABR
Annual mortality rate of general population at 45–85‐years‐old		0.002491–0.082156	
Annual mortality of progressive disease with chemotherapy plus BCS		0.6268 (0.4624–0.8105)	
Procedure‐related mortality rate at 45–85‐years‐old		0.0133–0.0800†	0.0037 (0–0.0208)
Procedure‐related mortality rate with conventional radiotherapy		0.0010	
One year probability of disease progression after primary treatment		0.0400 (0.0133–0.0742)	0.0790 (0.049–0.1284)
Rate of LR only/total recurrence		0	0.1844 (0.1451–0.4285)
Rate of RR only + LR&RR/total recurrence		0.2784 (0.0952–0.4)	0.1781 (0–0.3333)
The probability of radical salvage treatment for recurrence			
	Local failure only	0	0.2622 (0.2171–0.4865)
	Regional failure	0.3446 (0.3172–0.3888)	0.3010 (0.2941–0.3095)
	Distant failure	0	0
One year probability of disease progression after radical salvage treatment			
	In local recurrence	0	0.0679 (0–0.0799)
	In regional failure	0.2639 (0.2342–0.2865)	0.3115 (0.254–0.3835)

† 90‐day post‐treatment mortality rates of lobectomy.

Total 41 references for extracted parameters in Table 1 were attached to the supplement

BCS, best supportive care; LR, local recurrence; RR, regional recurrence; SABR, stereotactic ablative radiotherapy.

In the lobectomy group, patients with pathologically positive lymph node involvement were treated with adjuvant chemotherapy with or without radiotherapy. Regional recurrence was defined as recurrence at the stump, ipsilateral hilar, or the mediastinal lymph node. Some patients with regional recurrence were treated with salvage radiotherapy with or without chemotherapy. In the SABR group, regional recurrence was defined as recurrence at the ipsilateral hilar or mediastinal lymph node. Some patients with local recurrence were treated with salvage lobectomy, while patients with regional recurrence were treated with salvage radiotherapy with or without chemotherapy. In both groups, patients with recurrences who did not receive salvage treatment directly entered the state of disease progression. All patients with distant metastases directly entered the state of disease progression without salvage treatment (Fig 1).

A sensitivity analysis was conducted to explore the best strategies for overall survival by changing the variable values used in the model. A one‐way sensitivity analysis was performed to evaluate the effect of a single parameter (variable) while keeping the other parameters constant. A two‐way sensitivity analysis was performed to evaluate the effect of the simultaneous change in two variables. To evaluate the uncertainty associated with parameter estimation, we performed a second‐order Monte Carlo probabilistic sensitivity analysis.

### A systematic review of parameter estimation

We selected all articles published as abstracts or full papers in English from 2000 to September 2018 in peer‐reviewed journals that assessed a survival benefit or tumor response after lobectomy or SABR for the primary treatment for NSCLC less than 3 cm in size without lymph node or distant metastasis. Studies were identified by searching MEDLINE on PubMed using ‘lung cancer’ as the key text word in combination with ‘SABR’, ‘SBRT’, ‘stereotactic radiotherapy’, ‘surgery’ or ‘lobectomy’. The technique of lobectomy includes both video‐assisted thoracic surgery (VATS) and open lobectomy. The literature on SABR with low BEDs of less than 100 Gy or tumors located centrally were excluded. All estimates of the variables used in this model were obtained by a systematic review of published articles (Table 1). Whenever possible, estimates were extracted from meta‐analysis or randomized trials and, if not possible, from quasirandomized trials, prospective cohorts, and retrospective cohort studies in that order. A total of 41 references for the extracted parameters shown in Table 1 were attached to the supplement.

### Summary of parameters and assumptions

The age of the patients in the cohort was assumed to range from 45 to 85 years, and they were stratified into nine groups by fiveyear age windows at diagnosis (45, 50, 55, 60, 65, 70, 75, 80, and 85 years of age).

The procedure‐related mortality rate for lobectomy was 90 days. The post‐treatment mortality after surgery and parameters by age group were extracted from the Stokes’ study.^9^ For age groups not suggested in the study, we assumed the mortality rate by extrapolation. Since procedure‐related mortality rate for SABR is more affected by tumor location than age,^8^, ^9^ it was assumed that mortality rate was same for all age groups.

During follow‐up, some patients with locoregional recurrence were considered as candidates for salvage treatment. Although various treatment modalities could be applied for locoregional control, it was assumed that lobectomy would be performed for local recurrence after SABR, while radiotherapy would be performed for regional recurrence after lobectomy or SABR to simplify the Markov model. Solitary distant metastasis or secondary primary lung cancer also could be re‐treated with SABR or surgery. However, our tumor board determined that the likelihood of either of these procedures affecting the outcome would be low because the probability of occurrence is unknown, could fall within a very wide range and be similar in both groups.^3^, ^4^ As a result, all distant metastases were not considered as candidates for salvage treatment and secondary primary lung cancer was not reflected in this scenario to simplify the Markov model.

In the literature, 21–49% of patients with locoregional recurrence who had been treated with lobectomy or SABR for the primary NSCLC were treated with salvage treatment.^7^, ^14^, ^15^, ^16^, ^17^, ^18^, ^19^, ^20^ Theoretically, salvage treatment can be repeated two to three times in cases of recurrence. However, no articles demonstrating repeated salvage treatment for locoregional recurrence in NSCLC were found.^16^, ^20^, ^21^, ^22^, ^23^, ^24^, ^25^, ^26^, ^27^ Considering the clinical reality of locoregional recurrence, we limited the number of salvage treatments to just one.

Each outcome in the average was weighed by the number of patients in the articles to calculate an overall representative value for each component.

### Validation of the Markov model

We evaluated the validity of our Markov model by comparing the overall survival we obtained with those from previously reported studies that investigated NSCLC patients with tumors smaller than 3 cm. Predicted survival curves were created from our Markov model, and the survival outcomes of real studies were marked as points on the survival curves.

### Organization of the tumor board

For a systematic review of parameter estimation, validation of the Markov model and to discuss the overall scenario, we organized a small tumor board composed of a radiation oncologist, thoracic surgeon, and a pulmonologist.

## Results

### Predicted life expectancy

**Figure 2:**
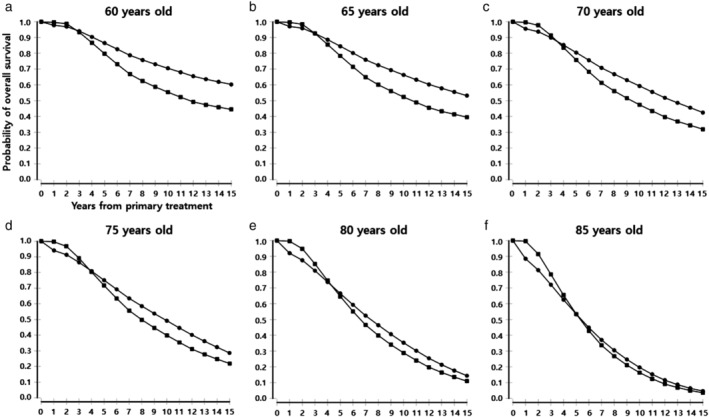
Estimated overall survival stratified by age at diagnosis in patients with stage IA NSCLC after lobectomy or SABR. Patients with stage IA NSCLC were stratified by age (**a**) 60 years, (**b**) 65 years, (**c**) 70 years, (**d**) 75 years, (**e**) 80 years, and (**f**) 85 years at diagnosis and overall survival was estimated in each of the cohorts using the Markov model. (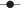) Lobectomy and (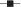) SABR.

The predicted life expectancies by five year age windows at diagnosis are shown in Table 2. The expected five year overall survival rates after lobectomy were found to be 90%, 89%, 88%, 86%, 84%, 80%, 75%, 66% and 53% at 45, 50, 55, 60, 65, 70, 75, 80, and 85 years of age at diagnosis, respectively. The expected five year overall survival rates after SABR were found to be 82%, 81%, 81%, 80%, 78%, 76%, 71%, 64% and 53% at 45, 50, 55, 60, 65, 70, 75, 80, and 85 years of age at diagnosis, respectively. Figure 2 shows the estimated overall survival curves for each cohort.

**Table 2:** Second‐order Monte Carlo simulation stratified by age: the difference of life expectancy between lobectomy and SABR

		Life expectancy (years)				
Age	Modality	Estimation	Mean difference	95% CI		*P*‐value
45	Lobectomy	12.60	1.69	0.89	2.43	0.0001
	SABR	10.91				
50	Lobectomy	12.43	1.68	0.93	2.47	0.0001
	SABR	10.75				
55	Lobectomy	12.22	1.60	0.83	2.43	0.0001
	SABR	10.62				
60	Lobectomy	11.82	1.47	0.67	2.31	0.0003
	SABR	10.35				
65	Lobectomy	11.35	1.32	0.52	2.17	0.0016
	SABR	10.03				
70	Lobectomy	10.50	1.00	0.18	1.75	0.0206
	SABR	9.49				
75	Lobectomy	9.43	0.72	−0.06	1.50	0.0689
	SABR	8.70				
80	Lobectomy	7.84	0.35	−0.37	1.03	0.3268
	SABR	7.49				
85	Lobectomy	6.07	−0.04	−0.64	0.53	0.8837
	SABR	6.12				

CI, confidence interval; SABR, stereotactic ablative radiotherapy.

### Second‐order Monte Carlo simulation

The mean differences and 95% confidence intervals (CIs) for the difference in the estimated life expectancies between the lobectomy and SABR groups are shown in Table 2. Lobectomy resulted in a better life expectancy than SABR, in patients under 75 years of age. However, the two procedures showed no statistically significant difference in patients 75 years or older. For 75‐year‐old patients, our model predicted life expectancies of 9.43 and 8.70 years following lobectomy and SABR, respectively, which was not significantly different. The 95% CI for the difference in life expectancies between lobectomy and SABR was ‐ 0.06–1.50 years at 75 years of age (*P* = 0.0689).

### One‐way sensitivity analysis

**Figure 3:**
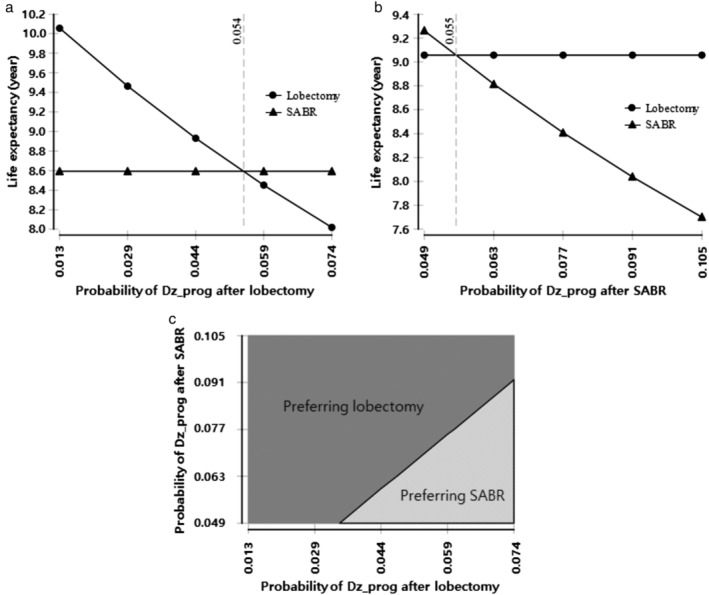
Sensitivity analysis of varying probability of disease progression after primary treatment in 75‐year‐old patients. One‐way sensitivity analysis of varying probability of disease progression after (**a**) lobectomy and (**b**) SABR. SABR could be a preferred strategy if the probability of these two variables were changed beyond the threshold. (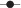) Lobectomy and (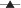) SABR. (**c**) Two‐way sensitivity analysis of the probability of disease progression after lobectomy and SABR: the dark gray region denotes lobectomy is preferred, while the light gray region shows SABR is preferred. SABR, stereotactic ablative radiotherapy; Dz_prog, disease progression.

A sensitivity analysis was performed for the group of 75‐year‐old patients. The tornado diagrams in Figure S1 show that life expectancy outcomes were most sensitive to the probability of disease progression after lobectomy or SABR. In contrast, life expectancy outcomes were less sensitive to variables related to treatment options such as procedure‐related mortality or local recurrence rate in SABR. For 75‐year‐old patients, our model predicted that lobectomy was the preferred strategy. However, the preferred strategy could be changed to SABR if the probability of disease progression after lobectomy was >0.054 (Fig 3a), or if the probability of disease progression after SABR was <0.055 (Fig 3b). Other variables did not alter the preferred treatment option from lobectomy.

### Two‐way sensitivity analysis

A two‐way sensitivity analysis demonstrated that the patients with a 1% probability of disease progression after lobectomy or SABR, had the same life expectancy when other variable values remained constant at preset values (Fig 3c). If the variables changed within the results of published studies as shown in Table 1, lobectomy was more likely to be selected as the treatment option at 75 years of age because the area of preferring lobectomy is wider than that for SABR. However, since SABR also could be a preferred strategy, the results are reversible.

### Model validity

**Figure 4:**
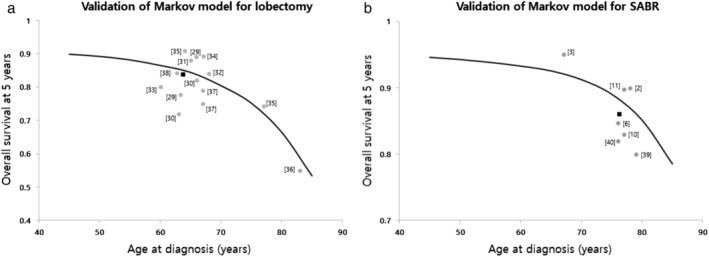
Validation of the Markov model. The predicted (**a**) fiveyear survival curve after lobectomy and (**b**) threeyear survival curve after SABR based on the Markov model are shown. The gray circles represent the survival outcomes from real studies, wherein the median age of each cohort was set to a representative value at diagnosis. The black square is the average of the values from these real studies. The mean overall survival following lobectomy and SABR in the real studies were approximately 1% and 2% lower, respectively than those obtained from the Markov model. SABR, stereotactic ablative radiotherapy. (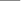) Markov model, (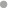) clinical trials, and (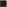) mean value of clinical trials.

Figure 4a illustrates the predicted five year survival rate after lobectomy. Outcomes from real studies are marked by gray dots.^28^, ^29^, ^30^, ^31^, ^32^, ^33^, ^34^, ^35^, ^36^, ^37^, ^38^ In real studies, the median age of each cohort was set to a representative value of age at diagnosis. A black square dot is the average of these real studies of lobectomy, and it is positioned very close to the survival curve of our Markov model. The mean five year overall survival in the real studies was about 1% lower than that observed in the Markov model. Figure 4b illustrates the predicted three year survival rate after SABR because almost studies on SABR have reported the three year overall survival rate. The outcomes of real SABR studies have been indicated using gray dots.^2^, ^3^, ^6^, ^10^, ^11^, ^39^, ^40^ A black square dot is the average of these real studies of SABR. The mean three year overall survival in the real studies was about 2% lower than that observed in the Markov model.

## Discussion

Lobectomy is undoubtedly the standard of care for operable early‐stage NSCLC in the current situation where there are no randomized trials comparing SABR with lobectomy. However, most of the previously reported studies on lobectomy included young patients with an average age of 65 years,^28^, ^29^, ^30^, ^31^, ^32^, ^33^, ^34^, ^35^, ^37^, ^38^ while those on SABR included older patients with an average age of 75 years.^1^, ^2^, ^4^, ^5^, ^6^, ^7^, ^10^, ^11^, ^12^, ^13^, ^14^ As a result, it is difficult to compare the overall survival between the two procedures directly using the existing research designs including patients of different age distributions. For medically operable patients with stage IA NSCLC, we conducted a virtual randomized phase III trial stratified by age using the Markov model analysis. Lobectomy resulted in a better life expectancy than SABR in patients under the age of 75 years, while no significant difference was observed in patients 75 years or older (Table 2). These results might be due to the rapid increase in surgical mortality seen in patients over 70 years of age. Lobectomy at age 70 years has a mortality rate approximately twice as high as that at age 60 years, and the risk increases with age.^9^, ^41^ These results are consistent with findings from actual clinical studies. Palm *et al.* used a population‐based registry to conduct a matched‐pair analysis of overall survival after surgery versus SABR for elderly patients (age > 75 years). Similar overall survival outcomes were achieved with surgery or SABR for stage I NSCLC in elderly patients. The three year overall survival has been reported to be 75% and 60% after surgery, and 87% and 42% after SBRT, respectively (log‐rank *P* = 0.22).^42^ Shirvani *et al.* also reported similar outcomes in the older age group. The Surveillance, Epidemiology, and End Results database linked to Medicare was used to determine the baseline characteristics and outcomes of 9093 patients with early‐stage, node‐negative NSCLC who underwent lobectomy or SABR. Propensity score‐matching analysis of well‐matched SABR and lobectomy cohorts demonstrated similar overall survival in both groups (adjusted hazard ratio, 1.01 [95% CI, 0.74–1.38]; P = 0.94). The median age in this study was 75 years.^43^ However, studies including younger age groups found surgery to be the better option. Recently, Wang *et al.* analyzed two clinical trials and seven cohort studies matched by propensity scores to compare the efficacy of SABR and lobectomy in stage I NSCLC. This meta‐analysis revealed significant benefits of lobectomy for three year overall survival (odds ratio, 2.11, 95% CI 1.55–2.86). It is noteworthy that most studies except one had included patients with a mean age of under 75 years.^44^ Bryant *et al.* reported the superiority of lobectomy by analysis of a large cohort (4069 patients) database from the Veteran's Affairs system. The multivariable analysis considering long‐term survival found higher cancer‐specific mortality associated with SABR compared with lobectomy (hazard ratio 1.45, 95% CI: 1.09–1.94, *P* = 0.01). The mean age of this cohort was also under 75 years.^45^

Unlike current studies involving only T1 tumors, previously terminated randomized trials (the STARS trial [NCT00840749] and the ACOSOG Z4099 trial [NCT01336894]) and the presently recruiting randomized trials (the SABRTooth trial [NCT02629458] the VALOR trial [NCT02984761]) included both T1 and T2 (AJCC eighth) tumors to compare the outcomes of surgical resection and SABR. When SABR is delivered with the planning target volume receiving BED more than 100 Gy, good local control is achieved in not only T1 but also T2 tumors. It results in local control in more than 90% of the tumors with very little acute or severe chronic toxicity.^3^, ^6^, ^12^ However, the benefit of sampling or dissection of mediastinal lymph nodes is expected to be higher in T2 tumors because of the higher nodal involvement in the more advanced T stages.^46^ After lobectomy for clinical N0 NSCLC, nodal upstaging was detected about two times at the clinical T2 stage compared to clinical T1 stage.^47^, ^48^, ^49^ The influence of tumor size on the clinical outcomes of SABR has been previously investigated in several studies.^1^, ^2^, ^5^, ^10^, ^11^, ^12^, ^13^ In a phase II study by Baumann *et al.* T2 lesions were associated with significantly increased local, regional, and distant recurrences compared to T1 lesions at three years (*P* = 0.02).^5^ With a study design included both T1 and T2 tumors, it may be difficult to demonstrate that there is no relevant difference in efficacy between SABR and lobectomy.

In the present study, both VATS and open lobectomy were allowed. Clinicians who are of the opinion that the VATS and open lobectomy have similar outcomes might find this scenario disadvantageous for the lobectomy group because VATS may be associated with lower mortality than open lobectomy. Several meta‐analyses have shown no significant difference in the overall survival between VATS and open lobectomy for early NSCLC.^50^ These data, however, are in conflict with the majority of original reports that comprise these studies, which have concluded that open lobectomy is superior to VATS lobectomy with regards to long‐term overall survival.^50^ Since more studies are needed to compare VATS and open lobectomy, it would be better to include both the techniques in the randomized study comparing the outcomes of lobectomy and SABR.

There are several limitations to this study. First, we simplified the scenario for the convenience of handling the Markov model. In a real clinical situation, solitary distant metastasis or secondary primary lung cancer can also be retreated with SABR or surgery, but the scenario of our model did not reflect them. We limited the number of salvage treatments to just one since we could not find any reports of repetitive salvage treatments, though several salvage treatments are possible in the clinic. Although simplification of the model may result in a discrepancy between the estimated values and reality, since the probability of these factors is identical for both groups, they would have little impact on clarifying the difference of survival in the two groups. Second, it was difficult to extract some parameters because of a lack of studies reporting their outcomes. There are only a few studies reporting the probability of disease progression after radical salvage treatment for regional recurrence in stage IA NSCLC patients treated with SABR or lobectomy. Therefore, we extracted those parameters from studies of stage I to IIIA NSCLC treated with SABR or lobectomy (Table 1). There is a possibility that the probability of disease progression after radical salvage treatment for regional recurrence after SABR may have been overestimated. Third, the selection bias could have affected the results because many parameters for the Markov model were extracted from many selected studies. As already mentioned in the methodology, we made an effort to reduce the bias by citing the representative value of each parameter as the average value from many papers, without quoting it as the value of a specific paper.

In conclusion, for medically operable patients with stage IA NSCLC, our Markov model has verified that lobectomy is a preferred strategy for patients younger than 75 years, while for those older than 75 years there is no significant difference in the expected overall survival between SABR and lobectomy. Until the results of the randomized study are available in the future, we hope that the results of the present study can serve as the background and rationale for clinicians to plan treatment modalities in elderly patients with stage IA NSCLC. In addition, we recommend that age should be considered when designing future randomized studies.

## Funding

There was no funding support.

## Disclosure

We declare no competing interests. All authors are in agreement with the content of the manuscript.

## Author contributions

Young‐Seok Seo and Hak Jae Kim contributed to the conception and design of the study. Young‐Seok Seo, Hak Jae Kim, Sun Mi Choi, and Samina Park were responsible for data acquisition. Young‐Seok Seo and Hak Jae Kim analyzed the data. Young‐Seok Seo, Hak Jae Kim, Hong Gyun Wu, Sun Mi Choi, and Samina Park interpreted the data. Young‐Seok Seo and Hak Jae Kim prepared the first draft of the manuscript.

## Supporting information


**Figure S1** One‐way sensitivity analysis of variables affecting survival following lobectomy versus SABR in 75‐year‐old patients. The tornado diagram shows that the probability of disease progression after lobectomy or SABR is an important factor that affects survival outcomes. SABR could be a preferred strategy if the probability of these two variables were changed beyond the threshold. Other variables did not change the preferred treatment option from lobectomy. SABR, stereotactic ablative radiotherapy; LR, local recurrence; RR, regional recurrence.

**Table S1** Estimated values of the variables used for the Markov model extracted from the literature.
