# Supplementary figures and images for: Lobectomy versus stereotactic ablative radiotherapy for medically operable patients with stage IA non‐small cell lung cancer: A virtual randomized phase III trial stratified by age
Source: Thorac Cancer. 2019 May 23;10(6):1489–99. doi: 10.1111/1759-7714.13103 (PMC6558457; doi:10.1111/1759-7714.13103)

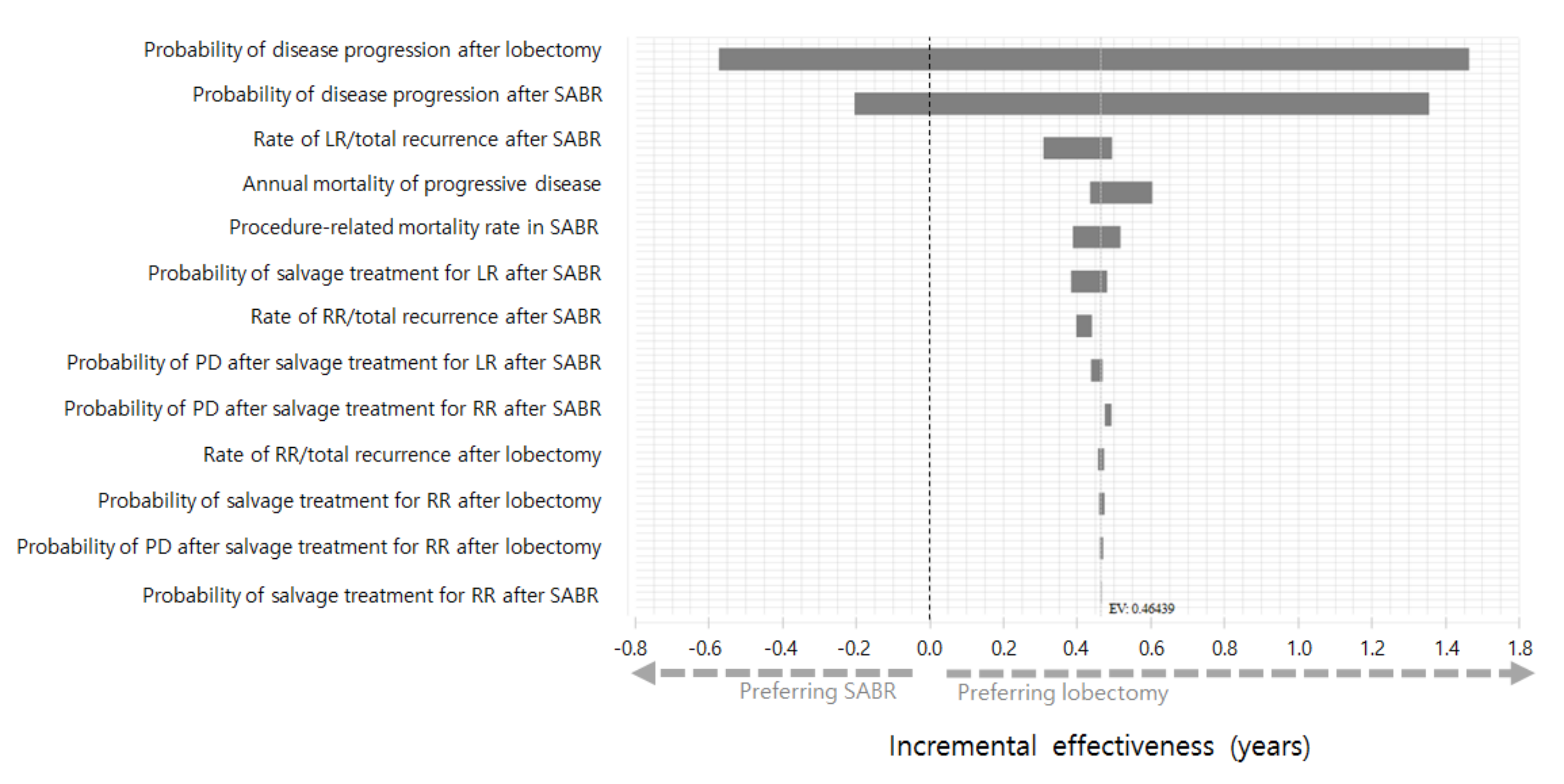

Supplement: Supplementary file 1 — Figure S1 One‐way sensitivity analysis of variables affecting survival following lobectomy versus SABR in 75‐year‐old patients. The tornado diagram shows that the probability of disease progression after lobectomy or SABR is an important factor that affects survival outcomes. SABR could be a preferred strategy if the probability of these two variables were changed beyond the threshold. Other variables did not change the preferred treatment option from lobectomy. SABR, stereotactic ablative radiotherapy; LR, local recurrence; RR, regional recurrence. [file TCA-10-1489-s001.png]
