# Supplementary material for: Lobectomy versus stereotactic ablative radiotherapy for medically operable patients with stage IA non‐small cell lung cancer: A virtual randomized phase III trial stratified by age
Source: Thorac Cancer. 2019 May 23;10(6):1489–99. doi: 10.1111/1759-7714.13103 (PMC6558457; doi:10.1111/1759-7714.13103)
Supplement: Supplementary file 2 — Table S1 Estimated values of the variables used for the Markov model extracted from the literature. [file TCA-10-1489-s002.docx]

| **Variables** | | **Lobectomy** | **SABR** |
| --- | --- | --- | --- |
| Annual mortality rate of general population at 45-85 years old | | 0.002491-0.082156^1^ | |
| Annual mortality of progressive disease with systemic therapy plus BCS | | 0.6268 (0.4624-0.8105)^2, 3^ | |
| Procedure-related mortality rate at 45-85 years old | | 0.0133-0.0800^*4^ | 0.0037 (0-0.0208)^5-14^ |
| Procedure-related mortality rate with conventional radiotherapy | | 0.0010^12^ | |
| 1-year probability of disease progression after primary treatment | | 0.0400 (0.0133-0.0742)^15-24^ | 0.0790 (0.049-0.1284)^5, 7, 8, 10, 11, 25^ |
| Rate of LR only/total recurrence | | 0 | 0.1844 (0.1451-0.4285)^5, 7, 26, 27^ |
| Rate of RR only+LR&RR/total recurrence | | 0.2784 (0.0952-0.4)^18, 19, 22, 24^ | 0.1781 (0-0.3333)^5, 7, 26, 27^ |
| Probability of radical salvage treatment for recurrence | |  |  |
|  | Local failure only | 0 | 0.2622 (0.2171-0.4865)^28-31^ |
|  | Regional failure | 0.3446 (0.3172-0.3888)^32, 33^ | 0.3010 (0.2941-0.3095)^26, 34^ |
|  | Distant failure | 0 | 0 |
| 1-year probability of disease progression after radical salvage treatment | |  |  |
|  | In local recurrence | 0 | 0.0679 (0-0.0799)^30, 35-37^ |
|  | In regional failure | 0.2639 (0.2342-0.2865)^38-40^ | 0.3115 (0.254-0.3835)^34, 41^ |

**Supplement_Table 1. Estimated values of the variables used for the Markov model extracted from the literature**

SABR; stereotactic ablative radiotherapy, BCS; best supportive care, LR; local recurrence, RR; regional recurrence

^*^90-day post-treatment mortality rates of lobectomy

References

1. Arias E, Heron M, Xu J. United States life tables, 2014. *National Vital Statistics Reports* 2017;66:1-63.

2. Noble J, Ellis PM, Mackay JA, et al. Second-line or subsequent systemic therapy for recurrent or progressive non-small cell lung cancer: a systematic review and practice guideline. *Journal of thoracic oncology : official publication of the International Association for the Study of Lung Cancer* 2006;1:1042-1058.

3. Zhong C, Liu H, Jiang L, et al. Chemotherapy plus best supportive care versus best supportive care in patients with non-small cell lung cancer: a meta-analysis of randomized controlled trials. *PloS one* 2013;8:e58466.

4. Stokes WA, Bronsert MR, Meguid RA, et al. Post-Treatment Mortality After Surgery and Stereotactic Body Radiotherapy for Early-Stage Non-Small-Cell Lung Cancer. *Journal of clinical oncology : official journal of the American Society of Clinical Oncology* 2018:Jco2017756536.

5. Koto M, Takai Y, Ogawa Y, et al. A phase II study on stereotactic body radiotherapy for stage I non-small cell lung cancer. *Radiotherapy and oncology : journal of the European Society for Therapeutic Radiology and Oncology* 2007;85:429-434.

6. van der Voort van Zyp NC, Prevost JB, Hoogeman MS, et al. Stereotactic radiotherapy with real-time tumor tracking for non-small cell lung cancer: clinical outcome. *Radiotherapy and oncology : journal of the European Society for Therapeutic Radiology and Oncology* 2009;91:296-300.

7. Takeda A, Sanuki N, Kunieda E, et al. Stereotactic body radiotherapy for primary lung cancer at a dose of 50 Gy total in five fractions to the periphery of the planning target volume calculated using a superposition algorithm. *International journal of radiation oncology, biology, physics* 2009;73:442-448.

8. Chang JY, Senan S, Paul MA, et al. Stereotactic ablative radiotherapy versus lobectomy for operable stage I non-small-cell lung cancer: a pooled analysis of two randomised trials. *The Lancet Oncology* 2015;16:630-637.

9. Nagata Y, Hiraoka M, Shibata T, et al. Prospective Trial of Stereotactic Body Radiation Therapy for Both Operable and Inoperable T1N0M0 Non-Small Cell Lung Cancer: Japan Clinical Oncology Group Study JCOG0403. *International journal of radiation oncology, biology, physics* 2015;93:989-996.

10. Sun B, Brooks ED, Komaki RU, et al. 7-year follow-up after stereotactic ablative radiotherapy for patients with stage I non-small cell lung cancer: Results of a phase 2 clinical trial. *Cancer* 2017;123:3031-3039.

11. Baumann P, Nyman J, Hoyer M, et al. Outcome in a prospective phase II trial of medically inoperable stage I non-small-cell lung cancer patients treated with stereotactic body radiotherapy. *Journal of clinical oncology : official journal of the American Society of Clinical Oncology* 2009;27:3290-3296.

12. Grutters JP, Kessels AG, Pijls-Johannesma M, et al. Comparison of the effectiveness of radiotherapy with photons, protons and carbon-ions for non-small cell lung cancer: a meta-analysis. *Radiotherapy and oncology : journal of the European Society for Therapeutic Radiology and Oncology* 2010;95:32-40.

13. Bryant AK, Mundt RC, Sandhu AP, et al. Stereotactic Body Radiation Therapy Versus Surgery for Early Lung Cancer Among US Veterans. *The Annals of thoracic surgery* 2018;105:425-431.

14. Fakiris AJ, McGarry RC, Yiannoutsos CT, et al. Stereotactic body radiation therapy for early-stage non-small-cell lung carcinoma: four-year results of a prospective phase II study. *International journal of radiation oncology, biology, physics* 2009;75:677-682.

15. Tsutani Y, Miyata Y, Nakayama H, et al. Oncologic outcomes of segmentectomy compared with lobectomy for clinical stage IA lung adenocarcinoma: propensity score-matched analysis in a multicenter study. *The Journal of thoracic and cardiovascular surgery* 2013;146:358-364.

16. Shiraishi T, Shirakusa T, Hiratsuka M, et al. Video-assisted thoracoscopic surgery lobectomy for c-T1N0M0 primary lung cancer: its impact on locoregional control. *The Annals of thoracic surgery* 2006;82:1021-1026.

17. Sakuraba M, Miyamoto H, Oh S, et al. Video-assisted thoracoscopic lobectomy vs. conventional lobectomy via open thoracotomy in patients with clinical stage IA non-small cell lung carcinoma. *Interactive cardiovascular and thoracic surgery* 2007;6:614-617.

18. Yoshino I, Yamaguchi M, Yamazaki K, et al. Surgical outcome of an anatomical resection of clinical stage IA non-small cell lung cancer assisted with a video-thoracoscopy. *Surgery today* 2010;40:719-724.

19. Maeda R, Yoshida J, Ishii G, et al. Long-term outcome and late recurrence in patients with completely resected stage IA non-small cell lung cancer. *Journal of thoracic oncology : official publication of the International Association for the Study of Lung Cancer* 2010;5:1246-1250.

20. Schuchert MJ, Kilic A, Pennathur A, et al. Oncologic outcomes after surgical resection of subcentimeter non-small cell lung cancer. *The Annals of thoracic surgery* 2011;91:1681-1687; discussion 1687-1688.

21. Schuchert MJ, Abbas G, Awais O, et al. Anatomic segmentectomy for the solitary pulmonary nodule and early-stage lung cancer. *The Annals of thoracic surgery* 2012;93:1780-1785; discussion 1786-1787.

22. Carr SR, Schuchert MJ, Pennathur A, et al. Impact of tumor size on outcomes after anatomic lung resection for stage 1A non-small cell lung cancer based on the current staging system. *The Journal of thoracic and cardiovascular surgery* 2012;143:390-397.

23. Yamashita S, Tokuishi K, Anami K, et al. Thoracoscopic segmentectomy for T1 classification of non-small cell lung cancer: a single center experience. *European journal of cardio-thoracic surgery : official journal of the European Association for Cardio-thoracic Surgery* 2012;42:83-88.

24. Kodama K, Higashiyama M, Takami K, et al. Treatment strategy for patients with small peripheral lung lesion(s): intermediate-term results of prospective study. *European journal of cardio-thoracic surgery : official journal of the European Association for Cardio-thoracic Surgery* 2008;34:1068-1074.

25. Lagerwaard FJ, Verstegen NE, Haasbeek CJ, et al. Outcomes of stereotactic ablative radiotherapy in patients with potentially operable stage I non-small cell lung cancer. *International journal of radiation oncology, biology, physics* 2012;83:348-353.

26. Senthi S, Lagerwaard FJ, Haasbeek CJ, et al. Patterns of disease recurrence after stereotactic ablative radiotherapy for early stage non-small-cell lung cancer: a retrospective analysis. *The Lancet Oncology* 2012;13:802-809.

27. Senthi S, Haasbeek CJ, Slotman BJ, et al. Outcomes of stereotactic ablative radiotherapy for central lung tumours: a systematic review. *Radiotherapy and oncology : journal of the European Society for Therapeutic Radiology and Oncology* 2013;106:276-282.

28. Verstegen NE, Lagerwaard FJ, Hashemi SM, et al. Patterns of Disease Recurrence after SABR for Early Stage Non-Small-Cell Lung Cancer: Optimizing Follow-Up Schedules for Salvage Therapy. *Journal of thoracic oncology : official publication of the International Association for the Study of Lung Cancer* 2015;10:1195-1200.

29. Allibhai Z, Cho BC, Taremi M, et al. Surgical salvage following stereotactic body radiotherapy for early-stage NSCLC. *The European respiratory journal* 2012;39:1039-1042.

30. Hamaji M, Chen F, Matsuo Y, et al. Treatment and Prognosis of Isolated Local Relapse after Stereotactic Body Radiotherapy for Clinical Stage I Non-Small-Cell Lung Cancer: Importance of Salvage Surgery. *Journal of thoracic oncology : official publication of the International Association for the Study of Lung Cancer* 2015;10:1616-1624.

31. Neri S, Takahashi Y, Terashi T, et al. Surgical treatment of local recurrence after stereotactic body radiotherapy for primary and metastatic lung cancers. *Journal of thoracic oncology : official publication of the International Association for the Study of Lung Cancer* 2010;5:2003-2007.

32. Hisakane K, Yoh K, Nakamura N, et al. Salvage chemoradiotherapy with cisplatin and vinorelbine for postoperative locoregional recurrence of non-small cell lung cancer. *Medicine* 2017;96:e8635.

33. Takenaka T, Takenoyama M, Toyozawa R, et al. Concurrent chemoradiotherapy for patients with postoperative recurrence of surgically resected non-small-cell lung cancer. *Clinical lung cancer* 2015;16:51-56.

34. Ward MC, Oh SC, Pham YD, et al. Isolated Nodal Failure after Stereotactic Body Radiotherapy for Lung Cancer: The Role for Salvage Mediastinal Radiotherapy. *Journal of thoracic oncology : official publication of the International Association for the Study of Lung Cancer* 2016;11:1558-1564.

35. Antonoff MB, Correa AM, Sepesi B, et al. Salvage pulmonary resection after stereotactic body radiotherapy: A feasible and safe option for local failure in selected patients. *The Journal of thoracic and cardiovascular surgery* 2017;154:689-699.

36. Mizobuchi T, Yamamoto N, Nakajima M, et al. Salvage surgery for local recurrence after carbon ion radiotherapy for patients with lung cancer. *European journal of cardio-thoracic surgery : official journal of the European Association for Cardio-thoracic Surgery* 2016;49:1503-1509.

37. Chen F, Matsuo Y, Yoshizawa A, et al. Salvage lung resection for non-small cell lung cancer after stereotactic body radiotherapy in initially operable patients. *Journal of thoracic oncology : official publication of the International Association for the Study of Lung Cancer* 2010;5:1999-2002.

38. Seol KH, Lee JE, Cho JY, et al. Salvage radiotherapy for regional lymph node oligo-recurrence after radical surgery of non-small cell lung cancer. *Thoracic cancer* 2017;8:620-629.

39. Kim E, Song C, Kim MY, et al. Long-term outcomes after salvage radiotherapy for postoperative locoregionally recurrent non-small-cell lung cancer. *Radiation oncology journal* 2017;35:55-64.

40. Lee NK, Moon SH, Kim TH, et al. Prognostic value of gross tumor volume for definitive radiation therapy in patients with locoregionally recurrent non-small-cell lung cancer after surgical resection. *Clinical lung cancer* 2013;14:399-406.

41. Kilburn JM, Lester SC, Lucas JT, Jr., et al. Management of mediastinal relapse after treatment with stereotactic body radiotherapy or accelerated hypofractionated radiotherapy for stage I/II non-small-cell lung cancer. *Journal of thoracic oncology : official publication of the International Association for the Study of Lung Cancer* 2014;9:572-576.
